# Triglyceride glucose index predicts long-term mortality and major adverse cardiovascular events in patients with type 2 diabetes

**DOI:** 10.1186/s12933-025-02671-2

**Published:** 2025-03-10

**Authors:** Matilde Sbriscia, Dalila Colombaretti, Angelica Giuliani, Silvia Di Valerio, Lucia Scisciola, Iryna Rusanova, Anna Rita Bonfigli, Fabiola Olivieri, Jacopo Sabbatinelli

**Affiliations:** 1 Scientific Direction, IRCCS INRCA, Ancona, Italy; 2 Department of Clinical and Molecular Sciences, Università Politecnica Delle Marche, Via Tronto 10/A, 60126 Ancona, Italy; 3 Cardiac Rehabilitation Unit of Bari Institute, Istituti Clinici Scientifici Maugeri IRCCS, Bari, Italy; 4 Department of Advanced Medical and Surgical Sciences, University of Campania “Luigi Vanvitelli”, Piazza L. Miraglia 2, 80138 Naples, Italy; 5 Departamento de Bioquímica y Biología Molecular I, Facultad de Ciencias, Instituto de Biotecnología, Parque Tecnológico de Ciencias de la Salud, Universidad de Granada, Granada, Spain; 6 Advanced Technology Center for Aging Research, IRCCS INRCA, Ancona, Italy; 7 Clinic of Laboratory and Precision Medicine, IRCCS INRCA, Ancona, Italy

**Keywords:** Type 2 diabetes, Triglyceride–glucose index, All-cause mortality, Major adverse cardiovascular events, Insulin resistance

## Abstract

**Background:**

The triglyceride glucose index (TyG index) is a marker of insulin resistance linked to the incidence of major adverse cardiovascular events (MACE) in diverse populations. However, its long-term prognostic role in type 2 diabetes (T2D) remains underexplored. This study evaluated the predictive value of the TyG index for all-cause mortality and MACE in T2D over a period of more than 15 years.

**Methods:**

A retrospective analysis was conducted on a cohort of 568 patients with T2D (median age: 67 years, IQR 61–72 years; 54% males; median disease duration: 14 years, IQR 7–21 years; median HbA1c: 7.3%, IQR 6.6–8.0%) and 376 presumably healthy controls (CTR, median age: 65 years, IQR 60–71 years) followed for a median period of 16.8 (IQR, 13.1–16.8) years. Routine biomarkers were measured on serum samples using commercially available methods. One-way ANOVA/ANCOVA, logistic regression, and Spearman’s correlations were used to compare the TyG index among groups and to assess its correlations with biochemical variables. The association between TyG index and the follow-up endpoints was investigated by Kaplan–Meier curves and Cox proportional hazards analysis.

**Results:**

Patients with T2D exhibited higher TyG Index values compared to CTR, with significant correlations between the TyG Index and markers of obesity, glucose metabolism, inflammation, and liver function. Patients with preexisting diabetic kidney disease (DKD) or atherosclerotic vascular disease had higher baseline values of TyG index. Sex-specific differences were observed among CTR but not in T2D patients. The TyG Index was predictive of all-cause mortality (HR = 1.39, 95% CI 1.07–1.79) and associated with the onset of complications MACE, DKD, and neuropathy independent of other conventional predictors. Age modified the TyG Index-mortality association, with the strongest effect in individuals aged 57–74.

**Conclusion:**

The TyG index is a valuable prognostic marker for long-term risk of all-cause mortality and MACE in patients with T2D, supporting its use in clinical risk stratification.

**Supplementary Information:**

The online version contains supplementary material available at 10.1186/s12933-025-02671-2.

## Introduction

Type 2 diabetes mellitus (T2D) is a chronic metabolic disorder characterized by elevated blood glucose levels due to insulin resistance (IR), a condition in which insulin-sensitive cells lose their ability to effectively respond to the hormone. Persistent, poorly managed hyperglycemia can lead to a range of micro- and macrovascular complications [1]. Among T2D-related complications, cardiovascular disease (CVD) represents the greatest cause of mortality, significantly surpassing other diabetes-related conditions in terms of associated death rates [2, 3]

IR is a significant factor in the pathogenesis of diabetes and can manifest 1–2 decades before T2D is formally diagnosed [4]. Moreover, IR exacerbates other cardiovascular risk factors, such as hypertension and dyslipidemia, further elevating the risk of adverse cardiovascular events and mortality in patients with T2D [5, 6]. Effective management of insulin resistance is crucial to reducing cardiovascular (CV) risk and improving survival outcomes in affected individuals.

The relationship between CVD and IR is complex, involving both metabolic and inflammatory dysfunctions. Therefore, finding a reliable marker for insulin resistance is of utmost importance for the effective management of diabetes and the prediction of its complications. Various methods are available to assess IR, including the homeostasis model assessment of insulin resistance (HOMA-IR) and the quantitative insulin sensitivity check index (QUICKI), which are commonly used in clinical practice. HOMA-IR is a widely used tool for estimating insulin resistance, but it has limitations in managing T2D. It assumes a constant relationship between insulin and glucose levels, which may not hold true for everyone, especially in advanced T2D, where β-cell function is impaired or in patients treated with insulin [7]. From a laboratory point of view, the insulin test, required for the calculation of HOMA-IR and QUICKI, is relatively expensive and shows poor reproducibility [7].

The triglyceride–glucose (TyG) index is a reliable surrogate marker of IR, combining fasting triglycerides and glucose levels, and it has now gained recognition as a potential predictor of adverse cardiovascular events [8]. The calculation of the TyG index is a simple, cost-effective tool that complements traditional markers, aiding in comprehensive diabetes care and improving long-term health outcomes. It is calculated as the logarithm of the product of fasting plasma triglyceride (TG) and fasting plasma glucose (FPG) levels, reflecting the interplay between lipid and glucose metabolism.

Recently, the TyG index has been associated with all-cause and cardiovascular mortality in the general population across diverse global populations during a 13.2-year follow-up. Findings indicate that higher TyG index levels are linked with the onset of T2D and an increased risk of major CV events and mortality, particularly in low- and middle-income countries [9]. Moreover, compared to HOMA-IR, the TyG index demonstrated superior predictive performance for all-cause and cardiovascular mortality [10]. However, fewer studies have investigated the prognostic value of the TyG index in patients with established T2D [11, 12], demonstrating that baseline TyG index values had the potential to predict both all-cause and CVD mortality in patients with T2D. Further research is needed to confirm the TyG index as a reliable predictor of T2D outcomes, especially for tracking the onset of non-cardiovascular complications and over longer follow-up periods.

This study aims to evaluate the association between the TyG index and long-term all-cause mortality and the development of T2D complications, including major adverse CV events (MACE), in a cohort of adults with T2D who were followed for over 16 years. The findings are expected to provide valuable insights into the utility of the TyG index as a risk-stratification tool for this population.

## Materials and methods

### Study population

The present study is a retrospective analysis of data from a cohort of 568 patients with T2D and 618 presumably healthy controls recruited at the Metabolic Diseases and Diabetology Department of IRCCS INRCA between May 2003 and November 2006 [13]. T2D was diagnosed according to the American Diabetes Association (ADA) criteria, which include one or more of the following: a hemoglobin A1c (HbA1c) level of ≥ 6.5%, a fasting blood glucose level of ≥ 126 mg/dl, a 2-h blood glucose level of ≥ 200 mg/dl following an oral glucose tolerance test (OGTT), or a random blood glucose level of ≥ 200 mg/dl in the presence of significant severe diabetes symptoms [14]. Inclusion criteria for patients in the T2D group were body mass index (BMI) of ≤ 40 kg/m^2^, age between 40 and 87 years, and the ability and willingness to provide written informed consent. Participants in the CTR group underwent a comprehensive medical assessment, including a detailed medical history and physical examination. Individuals with a known history of coronary artery disease, stroke, peripheral vascular disease, type 1 or 2 diabetes, and chronic kidney disease were excluded from the study.

The study was approved by the Institutional Review Board of IRCCS INRCA hospital (Approval No. 34/CdB/03). Written informed consent was obtained from each participant in accordance with the principles of the Declaration of Helsinki.

### Biomarkers

Fasting blood samples from all subjects were processed to obtain serum and stored at − 80 °C. Serum triglycerides and glucose were measured using commercially available blood chemistry assays on a Roche Cobas® c6000 chemistry analyzer. The TyG index was calculated as Ln [fasting triglycerides (mg/dL) × fasting plasma glucose (mg/dL)/2].

### Outcomes

Outcome events included the first occurrence of MACE (in participants without a prior history of MACE at enrollment), the development of T2D-related complications (in those without pre-existing complications at enrollment), and all-cause mortality. MACE was defined as a nonfatal event of myocardial infarction, cardiac arrest, cardiogenic shock, life-threatening arrhythmia, or stroke. Follow-up data on outcomes were obtained from medical records, covering the period from the date of enrollment (May 2003–November 2006) to the end of follow-up (December 31, 2019).

### Covariates

Data on vital signs, anthropometric measurements, medical history, lifestyle behaviors, physical activity, and concurrent treatments were collected from all participants. Blood cell counts and biochemical parameters were measured using standardized methods. The estimated glomerular filtration rate (eGFR) was determined using the CKD-EPI (Chronic Kidney Disease Epidemiology Collaboration) equation, which incorporates serum creatinine, age, sex, and ethnicity. Diabetic complications were identified as previously detailed [15]. Diabetic retinopathy was diagnosed through fundoscopy with dilated pupils and/or fluorescence angiography. Diabetic kidney disease (DKD) was characterized by a urinary albumin excretion rate exceeding 30 mg/24 h and/or eGFR < 60 mL/min/1.73m^2^. Neuropathy was diagnosed using electromyography. MACE was defined as non-fatal myocardial infarction or cerebrovascular accident. Peripheral artery disease, including atherosclerosis obliterans and cerebrovascular conditions, was identified through patient history, physical examination, and Doppler velocimetry.

### Statistical analysis

Continuous variables were presented as mean and standard deviation or as median and interquartile range, depending on their distribution, as evaluated using the Shapiro–Wilk test. Group comparisons of biomarkers were performed using the Mann–Whitney U test or the Kruskal–Wallis test with Dunn’s post-hoc analysis. Categorical variables were analyzed using the χ^2^ test. Spearman’s correlation was applied to examine relationships between continuous variables. Multivariable ANCOVAs were constructed with the TyG index as the dependent variable, T2D complications as factors, and age, sex, and HbA1c as covariates to identify factors associated with T2D complications, with univariate tests used for post-hoc comparisons. The association between the TyG index, modeled as a continuous or categorized variable, and follow-up outcomes was analyzed using Kaplan–Meier survival curves and Cox proportional hazards models, adjusted for sex, age, smoking status, hypertension, statin therapy, presence of T2D complications, BMI, HbA1c, blood lipids, eGFR, and hs-CRP, with 95% confidence intervals reported. The optimal TyG index cut-off for predicting survival in T2D patients was determined using the Evaluate Cutpoints R package [16]. The package calculates maximally selected rank statistics and estimates a cutpoint (the point with the most significant split based on the standardized log—rank test). A restricted cubic spline function was applied using the interactionRCS R package to assess the age-related relationship between TyG index and all-cause mortality. Logistic regression analyses were employed to assess associations with the onset of T2D complications, including MACE. Statistical significance was set at p < 0.05. All analyses were conducted using R (version 4.3), Jamovi software (version 2.4.1), and SPSS 28.0 for Windows (SPSS Inc., Chicago, IL, USA).

## Results

The analysed cohort includes 568 patients affected by T2D (median age = 67.0 years, IQR 56.0–92.0) and 376 presumably healthy controls (CTR; median age = 65.0, IQR 40.0–87.0). A general overview of patients’ baseline data is provided in Table 1. Among T2D subjects, 309 (54.4%) had at least one complication among retinopathy (27%), neuropathy (18%), MACE (15%), diabetic kidney disease (13%) and peripheral artery diseases (9%). During the follow-up, 159 patients (61.4%) of the 259 (45.6%) patients initially enrolled with uncomplicated T2D developed at least one complication. Subjects with T2D had higher triglyceride levels, BMI, weight, waist-hip ratio, hemoglobin (Hb), fasting glucose, HOMA-IR index, HbA1c, high-sensitivity C-reactive protein (hs-CRP) and white blood cells (WBC) than healthy subjects. LDL-C and total cholesterol levels were higher in CTR than in T2D patients, presumably due to the higher prevalence of statin therapy among T2D subjects (19.2% vs. 6.7%, p < 0.001). Moreover, healthy controls had significantly higher levels of platelets, aspartate aminotransferase (AST) and total bilirubin.

**Table 1:** Baseline characteristics of patients

Variable	CTR (N = 376)	T2D (N = 568)	p-value
TyG Index	8.38 (8.07–8.77)	9.11 (8.69–9.50)	** < 0.001**
Age	65 (60.0–71.0)	67 (61.0–72.0)	0.494
Sex (Males %)	147 (39%)	308 (54%)	** < 0.001**
Smoking (n, %)	54 (14%)	84 (15%)	0.847
Hypertension (n, %)	120 (32%)	364 (64%)	** < 0.001**
BMI (Kg/m^2^)	27 (24.6–29.7)	28.1 (25.8–31.4)	** < 0.001**
Weight (kg)	72 (64.0–81.0)	77 (69.0–86.0)	** < 0.001**
Waist-hip ratio	0.904 (0.847–0.950)	0.936 (0.893–0.980)	** < 0.001**
Total cholesterol (mg/dL)	218 (195–242)	206 (181–233)	** < 0.001**
LDL-C (mg/dL)	128 (108–149)	114 (95.5–135)	** < 0.001**
HDL-C (mg/dL)	56 (46–66)	50 (42–60)	** < 0.001**
Triglycerides (mg/dL)	94 (68–133)	116 (83–159)	** < 0.001**
ApoA1 (mg/dL)	175 (157–195)	163 (146–186)	** < 0.001**
ApoB (mg/dL)	103 (87–121)	100 (83.8–118)	0.176
Fasting insulin (mg/dL)	5.64 (3.80–7.84)	5.75 (3.69–8.72)	0.350
Fasting glucose (mg/dL)	93 (88–99)	153 (133–183)	** < 0.001**
HbA1c (%)	5.70 (5.50–5.90)	7.30 (6.60–8.03)	** < 0.001**
HOMA-IR	1.29 (0.850–1.82)	2.15 (1.39–3.59)	** < 0.001**
Hemoglobin (g/dL)	14 (13.3–14.9)	14.3 (13.4–15.2)	**0.008**
WBC (n/mm^3^)	5.95 (5.06–6.92)	*6.59* (5.55–7.56)	** > 0.001**
Platelets (n/mm^3^)	222 (190–264)	210 (179–252)	**0.002**
hs-CRP (mg/L)	1.94 (0.970–4.72)	2.49 (1.20–4.75)	**0.017**
Iron (µg/dL)	78 (63–100)	81 (64.8–96.3)	0.468
Ferritin (ng/dL)	87.5 (48.6–140)	88.7 (46.7–162)	0.132
Creatinine (mg/dL)	0.8 (0.7–0.9)	0.9 (0.7–1)	** < 0.001**
eGFR (ml/min)	82.9 (70.5–98)	80.9 (66.2–86.9)	**0.001**
Blood urea nitrogen (mg/dL)	38 (33–44)	38 (32–46)	0.968
Uric acid (mg/dL)	4.70 (4–5.50)	4.60 (4–5.50)	0.946
ALT (U/L)	36 (33–42)	39 (34–48)	** < 0.001**
AST (U/L)	21 (18–25)	20 (16–24)	** < 0.001**
Gamma-glutamyltransferase (U/L)	49 (41.8–59)	50 (39–62)	0.565
Total bilirubin (mg/dL)	0.7 (0.5–0.8)	0.6 (0.5–0.8)	**0.047**
Disease duration (years)	–	14 (7–21)	–
*T2D complications*			
MACE	–	84 (15%)	–
Diabetic kidney disease	–	74 (13%)	–
Neuropathy	–	103 (18%)	–
Retinopathy	–	156 (27.5%)	–
Peripheral vascular disease	–	53 (9%)	–
*T2D-relevant treatments*			
Metformin	–	207 (36%)	–
Sulphonylueares	–	273 (48%)	–
Insulin	–	101 (18%)	–
Glinides	–	13 (2%)	–
–	–	109 (19%)	–

Median (IQR) for continuous variables and n (%) for categorical variables. P value from Mann–Whitney test for continuous variables and from chi-squared tests of association for categorical variables. *TyG* Triglyceride–glucose index, *BMI* Body mass index, *LDL-C* Low-density lipoprotein cholesterol, *HDL-C* High-density lipoprotein cholesterol, *ApoA1* Apolipoprotein *A1 ApoB* Apolipoprotein B, *HbA1c* Glycated hemoglobin, *HOMA-IR* Homeostatic model assessment of insulin resistance, *WBC* White blood cell count, *hs-CRP* High-sensitivity C-reactive protein, *eGFR* Estimated glomerular filtration rate, *ALT* Alanine aminotransferase, *AST* Aspartate aminotransferase, *MACE* Major adverse cardiovascular events, *T2D* Type 2 diabetes.

**Fig. 1:**
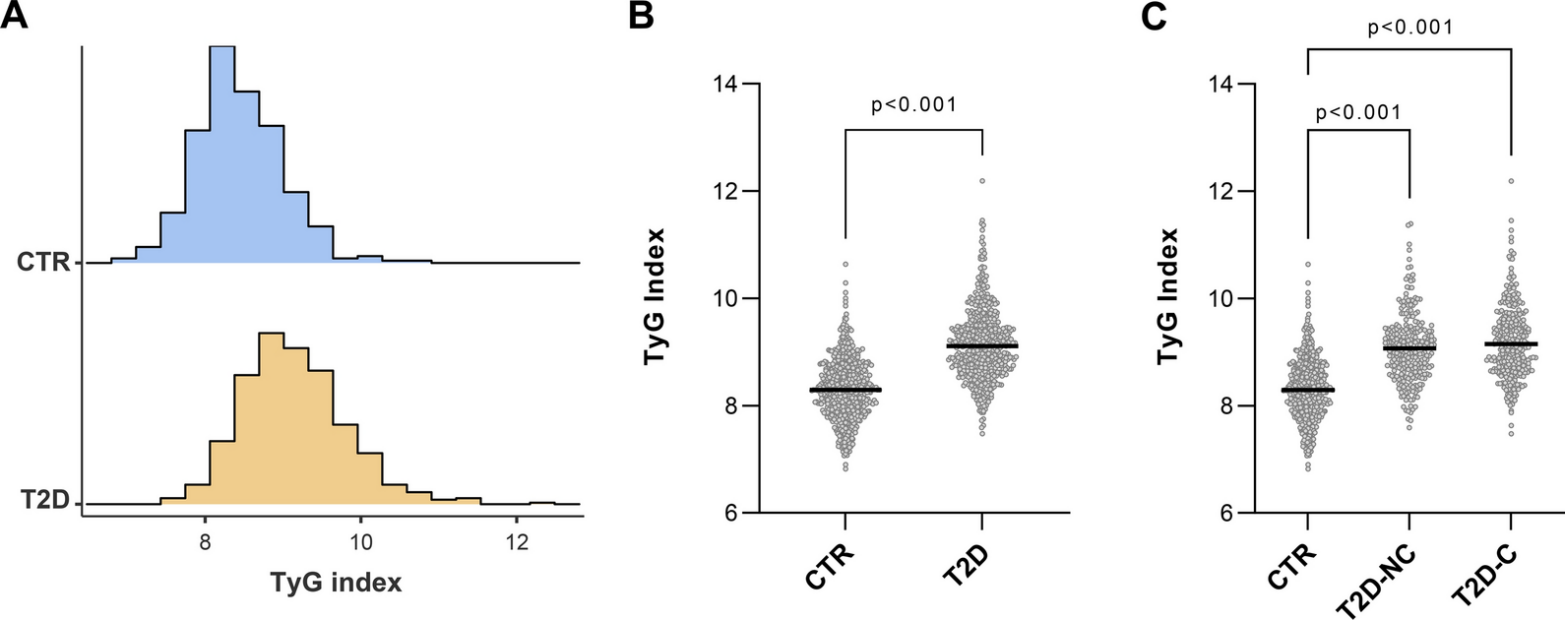
**A** Distribution of the TyG index in control (CTR) and type 2 diabetes (T2D) groups. **B** Comparison of TyG index between healthy controls (CTR) and patients with type 2 diabetes (T2D). Data are median and IQR. P-values for Mann–Whitney *U* test. **C** Distribution of TyG index among CTR and patients with uncomplicated (NC-T2D) or complicated (C-T2D) diabetes. P-values for Dunn’s post-hoc tests

The distribution of the TyG index differed significantly between the two study groups, with T2D patients showing a rightward shift compared to CTR, indicating higher values overall (Fig. 1A). When comparing mean values, subjects with T2D reported significantly higher TyG Index values than CTR (9.11 ± 0.69 vs 8.38 ± 0.56, p < 0.001) (Fig. 1B). T2D patients were also grouped into complicated (T2D-C) and non-complicated (T2D-NC) subgroups based on the presence of diabetes-related complications at enrollment. The discriminatory power of the TyG Index between T2D patients and healthy controls was confirmed by the Kruskal–Wallis test (p < 0.001), but post-hoc analysis did not reveal any significant difference due to T2D complications (p = 0.059, Fig. 1C). The sex-specific effect was also investigated, resulting in higher values of TyG index in men among controls (p < 0.001), while no significant differences were revealed in patients with T2D (p = 0.358) (Supplementary Fig. 1).

**Fig. 2:**
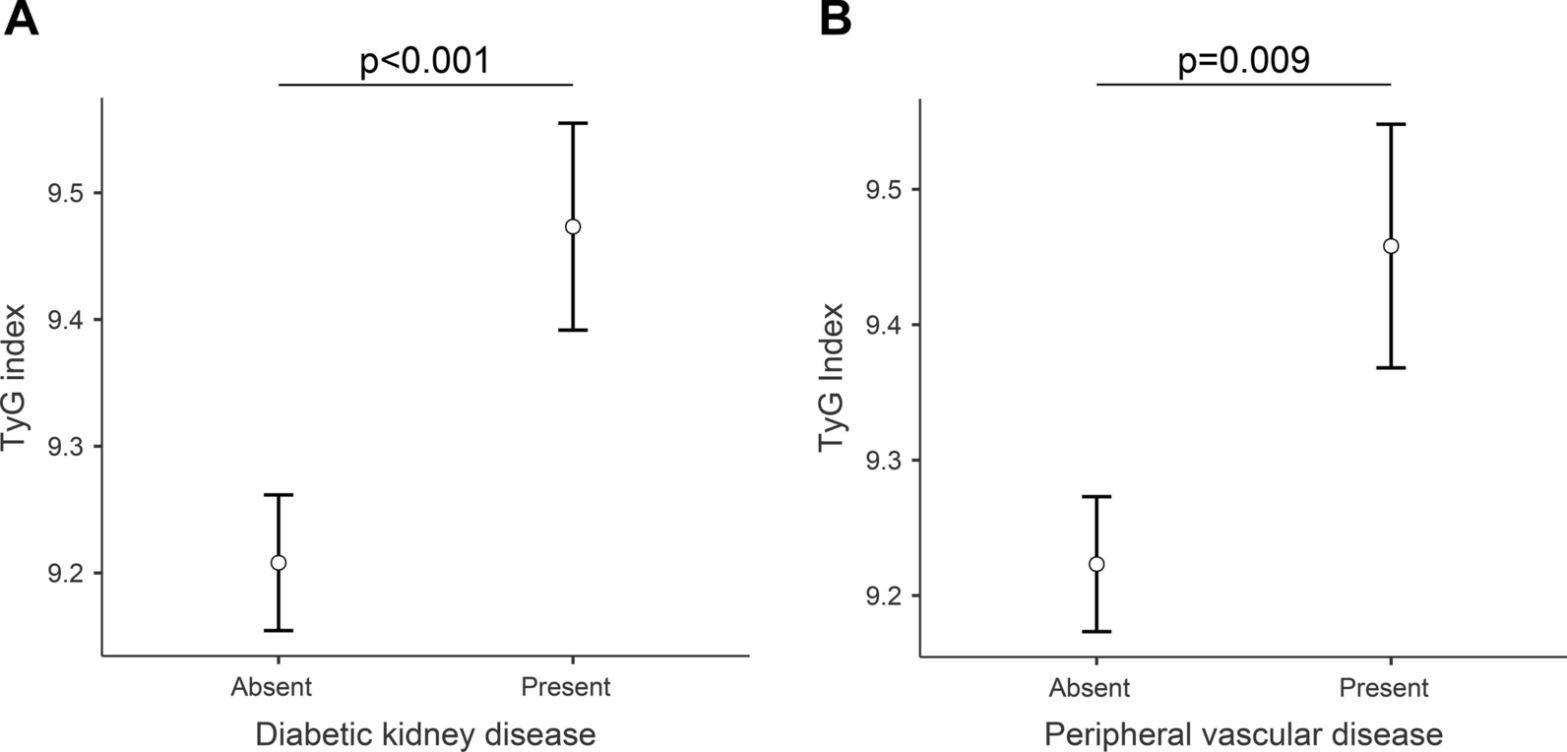
Comparison of TyG index in patients with or without **A** diabetic kidney disease or **B** peripheral vascular disease. Data are estimated marginal means with SEM. P values for post-hoc tests following ANCOVA (see text for details)

Differences in TyG index according to the presence of specific diabetes complications at baseline were also investigated. After adjustment for age, sex, HbA1c, and each other complication, the TyG index was higher in T2D patients with diabetic kidney disease (p < 0.001, Fig. 2A) or atherosclerotic vascular disease (p = 0.009, Fig. 2B), while no significant differences were registered for retinopathy, MACE, and neuropathy (Supplementary Table 1).

Moreover, we explored the TyG index correlations with the available biochemical data among CTR and T2D patients, reporting all Spearman’s coefficients in Supplementary Table 2. In both study groups, TyG Index was significantly correlated with BMI, weight and waist-hip ratio. Furthermore, a significant positive correlation is also evident with blood glucose control-related variables, such as HbA1c, fasting insulin and HOMA-IR. The TyG index plausible involvement in inflammatory processes is also supported by its positive correlation with WBC and hs-CRP in both T2D subjects and healthy controls, while IL-6 levels showed a significant positive correlation only in T2D patients. Regarding renal function, the TyG index displayed a negative correlation with eGFR and positive correlations with creatinine and uric acid levels. Additionally, the TyG Index positively correlates with liver function markers, such as alkaline phosphatase (ALP), alanine transaminase (ALT), gamma-glutamyl transferase (GGT). The lipid profile was also related with TyG index values, showing a negative correlation with HDL cholesterol and ApoA1, and a positive one with non-HDL cholesterol and ApoB. No significant correlation is reported between the TyG Index and cardiac biomarkers, such as N-terminal pro-B-type natriuretic peptide (NT-proBNP) and high-sensitivity troponin I, except for soluble ST2 (sST2), where the correlation with the TyG Index was slightly significant.

### Prognostic value of TyG index

**Fig. 3:**
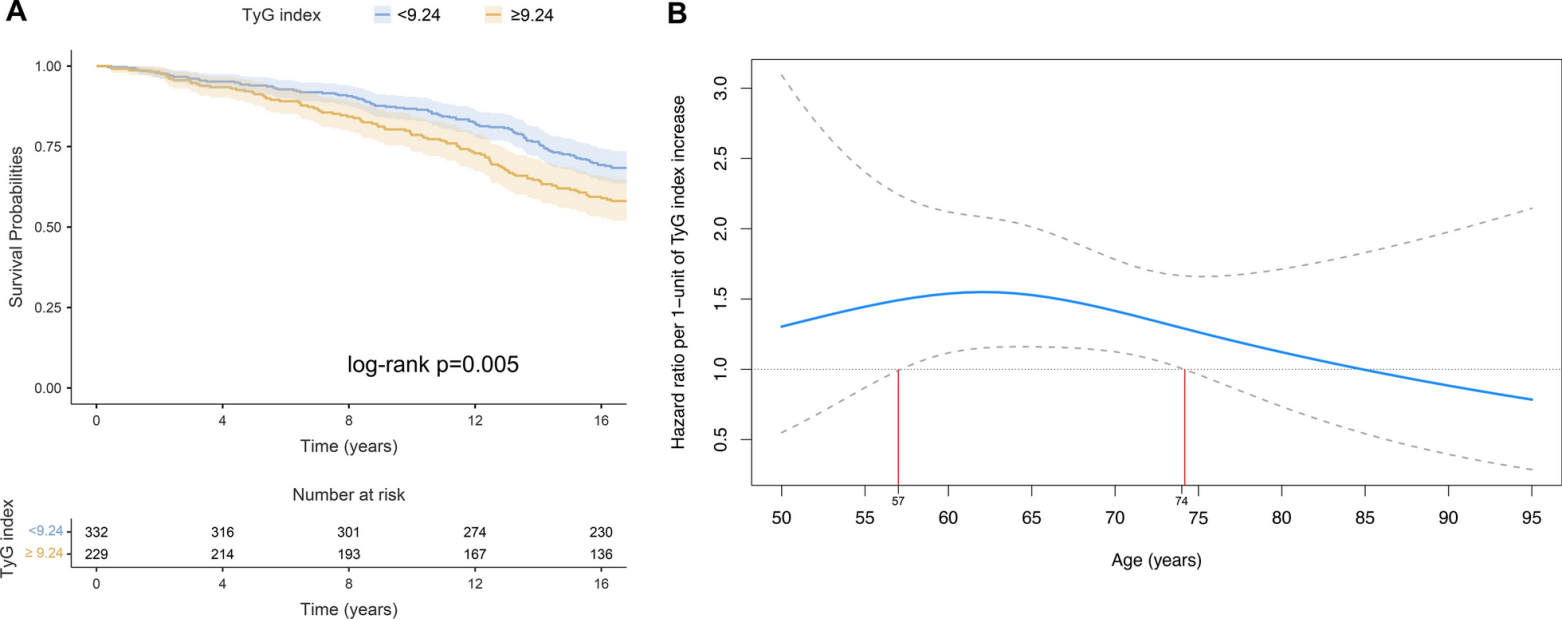
**A** Kaplan–Meier survival estimates for categorized TyG Index. **B** Restricted cubic spline used for evaluating the association between age and all-cause mortality according to TyG index. Hazard ratios were calculated using Cox proportional hazards regression analysis after adjusting for sex, smoking, hypertension, age, BMI, HbA1c, statin therapy, presence of T2D complications, eGFR, total cholesterol, and hs-CRP. Dashed lines are 95% confidence intervals. The horizontal dotted line at HR = 1.0 represents the null effect. The association between TyG and mortality is most pronounced around ages 57–74 years (highlighted by vertical red lines)

The predictive role of the TyG index in all-cause mortality and diabetes-related complications was assessed. T2D patients were followed for a median period of 16.8 years (IQR, 13.1–16.8). During this phase, 202 patients died (35.6%) and 7 patients (1.2%) were lost to follow-up, with a final mean survival time of 14.3 years (95% CI 13.9–14.7). Firstly, the TyG index prognostic relevance was evaluated using Cox regression methods. When TyG index was considered as a continuous variable, the univariate HR showed a significant correlation with all-cause mortality in T2D patients (HR = 1.30 (95% CI = 1.06–1.59); p = 0.013). Then, multiple Cox regression was performed, adjusting for the most relevant confounders (sex, smoking, hypertension, age, BMI, HbA1c, statin therapy, presence of T2D complications, eGFR, total cholesterol, and hs-CRP), obtaining a combined model that confirms the association between the increase of TyG index with higher mortality risk (HR = 1.33 (95% CI = 1.02–1.72); Table 2). Subsequently, the same statistical procedure was performed on the TyG index categorized using cutoffs obtained through an automated statistical procedure (see Methods for details) to maximize differences in survival prediction between groups (i.e. “Low” < 9.24; “High” ≥ 9.24). The adjusted Cox regression model (Table 2) and Kaplan–Meier survival analysis (Fig. 3A) confirmed an increased risk of mortality in subjects with a high TyG index.

**Table 2:** Univariable and multivariable Cox regression analysis for TyG Index (as continuous or categorized variable) as predictor of all-cause mortality

Model	Univariable	Multivariable	Univariable	Multivariable
Predictor	HR (95% CI)	HR (95% CI)	HR (95% CI)	HR (95% CI)
TyG_Index, continuous	**1.29 (1.05–1.59)**	**1.39 (1.07–1.79)**	–	–
TyG Index ≥ 9.24	–	–	**1.46 (1.11–1.93)**	**1.59 (1.14–2.17)**
Sex	1.23 (0.93–1.63)	**1.39 (1.03–1.87)**	1.21 (0.92–1.60)	**1.37 (1.02–1.86)**
Smoking	0.92 (0.61–1.37)	1.22 (0.81–1.85)	0.90 (0.60–1.37)	1.21 (0.80–1.83)
Hypertension	**1.52 (1.12–2.07)**	1.03 (0.74–1.43)	**1.52 (1.12–2.07)**	1.02 (0.74–1.41)
Statin therapy	1.16 (0.83–1.62)	0.83 (0.58–1.18)	1.16 (0.83–1.62)	0.84 (0.59–1.19)
T2D complications	**2.41 (1.78–3.26)**	**1.79 (1.28–2.48)**	**2.41 (1.78–3.26)**	**1.77 (1.27–2.46)**
Age	**1.10 (1.08–1.13)**	**1.11 (1.09–1.14)**	**1.10 (1.08–1.13)**	**1.11 (1.09–1.14)**
BMI	1.00 (0.97–1.03)	1.00 (0.97–1.04)	1.00 (0.97–1.03)	1.01 (0.97–1.04)
HbA1c	1.09 (0.99–1.21)	1.05 (0.92–1.19)	1.09 (0.99–1.21)	1.06 (0.94–1.20)
eGFR	**0.98 (0.97–0.99)**	0.99 (0.98–1.00)	0.98(0.97–0.99)	0.99 (0.98–1.00)
Total cholesterol	1.00 (1.00–1.00)	1.00 (0.99–1.00)	1.00 (1.00–1.00)	1.00 (0.99–1.00)
hs-CRP	**1.02 (1.01–1.04)**	**1.02 (1.01–1.04)**	**1.02 (1.01–1.04)**	**1.02 (1.00–1.04)**

Univariable and multivariable (adjusted for sex, smoking, hypertension, age, BMI, HbA1c, statin therapy, presence of T2D complications, eGFR, total cholesterol, and hs-CRP) hazard ratios (HR) with 95% confidence intervals (CI) are shown. Significant predictors in the analysis are in bold.

Conversely, no association was found between HOMA-IR and all-cause mortality (Supplementary Table 3).

Since some cases of T2D primarily involve reduced insulin secretion, we analyzed the association between the TyG index and all-cause mortality in individuals not receiving insulin therapy who had fasting insulin levels below the median (< 5.54 µIU/mL, n = 230). The continuous TyG index remained associated with mortality (HR = 1.36, 95% CI 0.86–2.13), with an effect size comparable to that observed in the full cohort. However, the association did not reach statistical significance after adjustment, likely due to limited sample size.

Then, as previous studies have reported variable associations of the TyG index with outcomes depending on age [17], we examined the relationship between TyG index and all-cause mortality across different age groups using a restricted cubic spline function. The results indicated that the association between the TyG index, measured continuously, and mortality was significant between the ages of 57 and 74 years (Fig. 3B). In individuals younger than 57 years, the association was present but not statistically significant, likely due to the limited sample size in this subgroup. Beyond 74 years of age, the association weakened, with the HR declining below 1.0, and the confidence intervals expanding, indicating no significant relationship between the TyG index and mortality in older individuals.

Finally, to evaluate the role of the TyG index as a predictor of T2D-related complications, we examined its association with the development of at least one complication in patients with uncomplicated diabetes at baseline. Multivariable logistic regressions were performed considering the same confounders of the previous analyses (sex, smoking, hypertension, age, BMI, HbA1c, statin therapy, presence of T2D complications, eGFR, total cholesterol, and hs-CRP). The resulting data revealed associations between the TyG index and the development of MACE (p = 0.045), neuropathy (p = 0.038) and diabetic kidney disease (p = 0.037) in patients with no history of these complications before the enrolment, while no significant association was found between TyG index and development of retinopathy or atherosclerotic vascular disease (Table 3).

**Table 3:** Multivariable logistic regression for the development of T2D complications

Outcome	TyG index OR (95% CI)	p-value
MACE	1.46 (1.02–2.10)	**0.046**
Peripheral vascular disease	1.17 (0.77–1.78)	0.469
Diabetic kidney disease	1.56 (1.04–2.36)	**0.033**
Neuropathy	1.57 (1.03–2.41)	**0.037**
Retinopathy	0.73 (0.46–1.14)	0.166

Odds ratios (OR) for 1-unit of TyG index increase. Model adjusted for sex, smoking, hypertension, age, BMI, HbA1c, statin therapy, presence of other T2D complications, eGFR, total cholesterol, and hs-CRP. Significant ^p^-values are in bold.

## Discussion

Our study provides comprehensive insights into the relationship between the TyG index, a marker of insulin resistance, and its association with all-cause mortality and diabetes-related complications in individuals with T2D, over a 16.9-year follow-up.

The prognostic significance of the TyG index for all-cause mortality was a central finding of the study. Cox regression analyses revealed that higher TyG index values were independently associated with an increased risk of mortality (HR = 1.33; 95% CI 1.02–1.72) after adjusting for relevant confounders. When stratified by a TyG index threshold of 9.24, higher values consistently predicted an increased risk of mortality. While this cutoff is slightly higher than those proposed for the general population [10, 18], it aligns well with thresholds identified as high risk in specific disease populations [19, 20]. Notably, age-specific analyses highlighted a significant relationship between the TyG index and mortality among individuals aged 57–74 years, while this association weakened in younger and older age groups, likely reflecting the complex interplay of competing risks and sample size variability over the extended follow-up.

Given that some cases of T2D primarily involve impaired insulin secretion rather than insulin resistance [21], we further explored the association between the TyG index and mortality in individuals not on insulin therapy who had fasting insulin levels below the median. While the TyG index retained a considerable effect size in its association with mortality, the relationship did not reach statistical significance, likely due to the limited sample size. This finding suggests that the TyG index may still capture relevant metabolic risk even in individuals with lower insulin levels, but its prognostic strength may vary across different T2D phenotypes.

Our study reinforced the association between the TyG index and biochemical markers commonly associated with diabetes, such as HbA1c, fasting insulin, HOMA-IR, eGFR, WBC, hs-CRP, IL-6, and liver function markers, which is consistent with previous studies [22–24]. These findings underscore the TyG index’s ability to accurately reflect both altered glycemic profiles and chronic inflammatory states, highlighting its potential for wider application in diabetes management.

When considering T2D complications, we observed higher TyG index values in subjects with ASCVD and DKD, while no difference was recorded in patients with history of MACE, probably due to aggressive lipid-lowering in these subjects. Lastly, the TyG index demonstrated predictive value for diabetes-related complications during the follow-up period. Higher TyG index values were linked with an increased risk of developing MACE, neuropathy, and diabetic kidney disease in patients without a prior history of these conditions. These findings are all consistent with previous literature, which has shown the ability of the TyG index to predict the development of MACE over shorter follow-up periods [25]. Moreover, our study extends the associations between the TyG index and diabetic kidney disease, and diabetic neuropathy [25, 26], which were previously observed only in cross-sectional studies, by demonstrating these links in a longitudinal design.

The influence of age on the TyG index’s ability to predict all-cause mortality warrants attention. In our cohort, the strongest association between the TyG index and all-cause mortality was observed in individuals aged 57–74 years. This may partly reflect the limited representation of younger patients in the study. Furthermore, older patients often have more comorbidities and poorer organ function, which may outweigh the prognostic value of the TyG index for mortality in this group of subjects with T2D. Supporting this, Cheng et al. demonstrated a robust association between the TyG index and mortality within a narrower age range of 45–64 years, highlighting the age-specific variability of the index’s predictive power in the general population [10]. Similarly, Yao et al. found that the TyG index’s predictive validity for all-cause and non-CV mortality persisted only in T2D patients younger than 65 years [12].

In contrast, Liu et al., while not performing age stratification, focused on young American patients with diabetes. Their findings revealed a non-linear association between TyG index values above 9.18 and all-cause mortality in individuals under 65 years [20]. Interestingly, Shen et al. reported a different trend in their ten-year follow-up, demonstrating that the TyG index predicts all-cause mortality in patients with acute coronary syndrome and T2D aged ≥ 80 years, extending its relevance to older populations [26].

Conflicting results across studies may stem from variations in cohort characteristics, comorbidities, and study methodologies, highlighting the impact of heterogeneity in study design. For example, Kim et al. observed a loss of predictive value for the TyG index in multivariable-adjusted models within the Korean population [27], and Chen et al. found no significant association between the TyG index and all-cause or cardiovascular mortality in the general American population [28].

Overall, these findings underscore significant age-related variability in the TyG index’s prognostic utility. While middle-aged and younger populations show stronger associations, older adults may present competing risks that dilute its predictive power.

A 2022 meta-analysis by Liu et al. of 32 studies highlighted the TyG index’s relationship with cardiac events and mortality in the general population, identifying it as a strong predictor of cardiovascular diseases, such as hypertension and atherosclerosis, particularly in T2D patients [29]. However, the analysis lacked definitive evidence confirming a significant association between the TyG index and all-cause mortality. In specific populations, such as T2D patients with established ASCVD, the predictive role of the TyG index appears more pronounced [30].

Although not fully understood, the TyG index’s association with mortality may be linked to its role as a marker of insulin resistance and its strong correlation with systemic inflammation, oxidative stress, and endothelial dysfunction. Its involvement in pathways contributing to atherosclerosis, organ dysfunction, and related complications underscores its potential as a comprehensive predictor of adverse outcomes, linking metabolic imbalance to increased mortality risk.

Our study presents some limitations. First, the retrospective design limits our ability to infer causality between the TyG index and mortality or T2D-related complications. Second, the lack of intermediate data on glycemic control, biochemical markers of T2D complications (e.g., urinary albumin-to-creatinine ratio), and changes in the TyG index during the follow-up period restricts insights into the relationship between these variables and outcomes. In particular, the absence of longitudinal TyG data prevents us from evaluating how its changes over time may influence the risk of diabetic complications and mortality. Third, while we observed a significant association between the TyG index and MACE, the lack of information on event timing prevented us from performing Cox regression analysis, limiting our ability to assess the temporal relationship between the TyG index and MACE risk. Fourth, while the long follow-up period strengthens the analysis, the progressive adoption of newer antidiabetic medications and evolving management strategies for glycemic and lipid control during this time may have influenced the results. Despite these limitations, the dedicated follow-up of T2D patients, adherence to contemporary care standards, and the use of preserved serum samples for biomarker analysis provide robustness to the findings. Future studies with longitudinal designs and comprehensive intermediate data collection are necessary to validate and extend these results.

To the best of our knowledge, this is the first study to emphasize the significant role of the TyG index in predicting all-cause mortality and T2D-related complications in patients with diabetes. Our analysis fills a gap in long-term research on this topic by utilizing an extensive follow-up period of 16.9 years. In this cohort, the TyG index demonstrates superior discriminatory ability for adverse outcomes compared to HOMA-IR, underscoring its potential as a valuable diagnostic tool in clinical practice. Importantly, the TyG index offers a cost-effective and practical tool for application in large population studies. Unlike HOMA-IR, which requires insulin assays that are characterized by higher costs, limited availability in many settings, and additional preanalytical challenges, the TyG index is based on readily available clinical parameters, making it a more accessible option for widespread use. Integrating TyG index measurements into routine check-ups could enhance early detection of complications and bolster preventive care efforts. Regular monitoring of TyG index trends may refine risk stratification and support targeted interventions to mitigate insulin resistance, ultimately reducing mortality risk.

## Supplementary Information

Supplementary Materials 1.

## Data Availability

The datasets generated during and/or analyzed during the current study are available from the corresponding author on reasonable request.
