# Supplementary material for: Triglyceride glucose index predicts long-term mortality and major adverse cardiovascular events in patients with type 2 diabetes
Source: Cardiovasc Diabetol. 2025 Mar 10;24:115. doi: 10.1186/s12933-025-02671-2 (PMC11895143; doi:10.1186/s12933-025-02671-2)
Supplement: Supplementary file 1 — Supplementary Materials 1. [file 12933_2025_2671_MOESM1_ESM.pdf]

Triglyceride glucose index predicts long-term mortality and major adverse cardiovascular events in patients with type 2 diabetes

SUPPLEMENTARY MATERIAL

Supplementary Figure 1. Sex-related comparison of TyG index in **(A)** healthy controls (CTR) and **(B)** patients with type 2 diabetes (T2D). Data are median and IQR. P-values for Mann–Whitney *U* test.

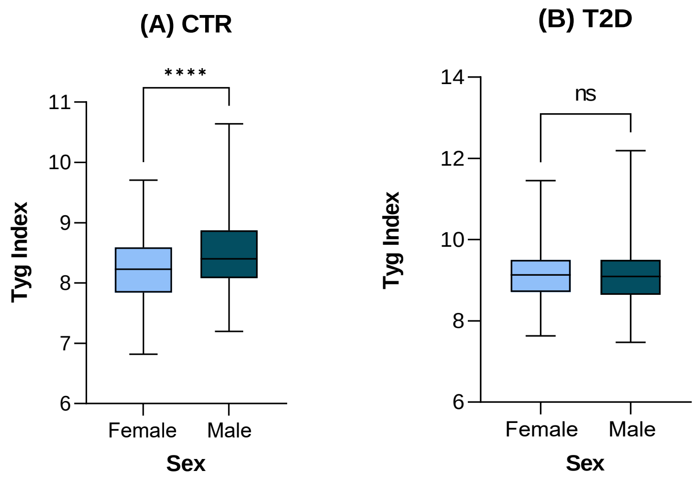

Supplementary Table 1. Estimated marginal means of TyG in T2D patients with specific complications.

	TyG index		
**Complication**	Present	Absent	p-value
MACE	9.387 (0.077)	9.320 (0.056)	0.349
Atherosclerotic vascular disease	9.477 (0.089)	9.230 (0.049)	**0.009**
Diabetic kidney disease	9.490 (0.081)	9.217 (0.053)	**< 0.001**
Neuropathy	9.360 (0.073)	9.347 (0.058)	0.847
Retinopathy	9.306 (0.064)	9.401 (0.064)	0.109

Data are estimated marginal means (SEM). Model adjusted for age, sex, and HbA1c. Significant p-values are in bold.

Supplementary Table 2. Spearman's correlation coefficients between the TyG index and the available biochemical variables.

Variable		Overall (n=944)	CTR (n=376)	T2D (n=568)
**Age**	Correlation coefficient<br>P-value	-0.010<br>0.750	0.115<br>**0.026**	-0.089<br>**0.034**
**BMI**	Correlation coefficient<br>P-value	0.330<br>**<.001**	0.295<br>**<.001**	0.286<br>**<.001**
**Weight**	Correlation coefficient<br>P-value	0.311<br>**<.001**	0.300<br>**<.001**	0.230<br>**<.001**
**HbA1c**	Correlation coefficient<br>P-value	0.583<br>**<.001**	0.241<br>**<.001**	0.421<br>**<.001**
**eGFR**	Correlation coefficient<br>P-value	-0.215<br>**<.001**	-0.229<br>**<.001**	-0.172<br>**<.001**
**BUN**	Correlation coefficient<br>P-value	-0.055<br>0.089	-0.045<br>0.388	-0.070<br>0.094
**Creatinine**	Correlation coefficient<br>P-value	0.261<br>**<.001**	0.232<br>**<.001**	0.156<br>**<.001**
**Uric acid**	Correlation coefficient<br>P-value	0.199<br>**<.001**	0.335<br>**<.001**	0.164<br>**<.001**
**RBC**	Correlation coefficient<br>P-value	0.088<br>**0.007**	0.158<br>**0.002**	0.047<br>**0.266**
**Hemoglobin**	Correlation coefficient<br>P-value	0.091<br>**0.005**	0.133<br>**0.010**	0.010<br>0.804
**Hematocrit**	Correlation coefficient<br>P-value	0.041<br>0.211	0.149<br>**0.004**	0.007<br>0.859
**MCV**	Correlation coefficient<br>P-value	-0.107<br>**<.001**	-0.045<br>0.381	-0.086<br>**0.041**
**MCH**	Correlation coefficient<br>P-value	0.015<br>0.649	-0.047<br>0.366	-0.058<br>0.168
**MCHC**	Correlation coefficient<br>P-value	0.158<br>**<.001**	-0.006<br>0.905	-0.006<br>0.892
**RDW-CV**	Correlation coefficient<br>P-value	-0.048<br>0.142	0.071<br>0.171	-0.019<br>0.649
**PLT**	Correlation coefficient<br>P-value	-0.006<br>0.856	0.077<br>0.135	0.036<br>0.390
**PLCR**	Correlation coefficient<br>P-value	-0.031<br>0.354	-0.142<br>**0.007**	0.020<br>0.637
**WBC**	Correlation coefficient<br>P-value	0.254<br>**<.001**	0.189<br>**<.001**	0.137<br>**0.001**
**Neutrophils (#)**	Correlation coefficient<br>P-value	0.241<br>**<.001**	0.210<br>**<.001**	0.151<br>**<.001**
**Lymphocytes (#)**	Correlation coefficient<br>P-value	0.152<br>**<.001**	0.175<br>**<.001**	0.110<br>**0.009**
**Basophils (#)**	Correlation coefficient<br>P-value	0.046<br>0.156	0.009<br>0.868	0.061<br>0.144
**Monocytes (#)**	Correlation coefficient<br>P-value	0.112<br>**<.001**	0.174<br>**<.001**	0.097<br>**0.020**
**Eosinophils (#)**	Correlation coefficient<br>P-value	0.075<br>0.021	0.104<br>0.045	0.015<br>0.721
**Cholesterol**	Correlation coefficient<br>P-value	0.091<br>**0.005**	0.178<br>**<.001**	0.228<br>**<.001**
**LDL-C**	Correlation coefficient<br>P-value	-0.053<br>0.103	0.090<br>0.084	0.016<br>0.705
**HDL-C**	Correlation coefficient<br>P-value	-0.498<br>**<.001**	-0.534<br>**<.001**	-0.449<br>**<.001**
**non-HDL-C**	Correlation coefficient<br>P-value	0.293<br>**<.001**	0.409<br>**<.001**	0.414<br>**<.001**
**Cholesterol remnant**	Correlation coefficient<br>P-value	0.496<br>**<.001**	0.520<br>**<.001**	0.577<br>**<.001**
**Triglycerides**	Correlation coefficient<br>P-value	0.870<br>**<.001**	0.978<br>**<.001**	0.896<br>**<.001**
**hs-CRP**	Correlation coefficient<br>P-value	0.261<br>**<.001**	0.222<br>**<.001**	0.289<br>**<.001**
**Fasting insulin**	Correlation coefficient	0.279	0.339	0.305

	P-value	**< .001**	**< .001**	**< .001**
**HOMA-IR**	Correlation coefficient<br>P-value	0.560<br>**< .001**	0.376<br>**< .001**	0.508<br>**< .001**
**ALP**	Correlation coefficient<br>P-value	0.139<br>**< .001**	0.104<br>**0.045**	0.194<br>**< .001**
**AST**	Correlation coefficient<br>P-value	0.227<br>**< .001**	0.098<br>0.058	0.121<br>**0.004**
**ALT**	Correlation coefficient<br>P-value	0.202<br>**< .001**	0.181<br>**< .001**	0.197<br>**< .001**
**GGT**	Correlation coefficient<br>P-value	0.309<br>**< .001**	0.319<br>**< .001**	0.360<br>**< .001**
**Total bilirubin**	Correlation coefficient<br>P-value	-0.102<br>**0.002**	-0.077<br>0.142	-0.092<br>**0.028**
**LDL-C**	Correlation coefficient<br>P-value	-0.053<br>0.103	0.090<br>0.084	0.016<br>0.705
**ApoA1**	Correlation coefficient<br>P-value	-0.282<br>**< .001**	-0.285<br>**< .001**	-0.186<br>**< .001**
**ApoB**	Correlation coefficient<br>P-value	0.199<br>**< .001**	0.276<br>**< .001**	0.265<br>**< .001**
**Serum iron**	Correlation coefficient<br>P-value	0.005<br>0.889	-0.038<br>0.464	-0.001<br>0.979
**Transferrin**	Correlation coefficient<br>P-value	0.158<br>**< .001**	0.143<br>**0.006**	0.095<br>**0.024**
**Ferritin**	Correlation coefficient<br>P-value	0.102<br>**0.002**	0.096<br>0.065	0.067<br>0.110
**Total protein**	Correlation coefficient<br>P-value	0.120<br>**< .001**	0.038<br>0.464	0.104<br>**0.014**
**hs-CRP**	Correlation coefficient<br>P-value	0.261<br>**< .001**	0.222<br>**< .001**	0.289<br>**< .001**
**IL-6**	Correlation coefficient<br>P-value	0.118<br>**0.018**	0.128<br>0.159	0.197<br>**< .001**
**sST2**	Correlation coefficient<br>P-value	0.142<br>**< .001**	0.027<br>0.716	0.084<br>**0.045**
**hs-cTnI**	Correlation coefficient<br>P-value	-0.019<br>0.615	0.014<br>0.868	-0.014<br>0.742
**NT-proBNP**	Correlation coefficient<br>P-value	-0.094<br>**< .001**	-0.013<br>0.880	-0.044<br>0.290
**Telomere length**	Correlation coefficient<br>P-value	-0.059<br>0.075	0.026<br>0.621	-0.040<br>0.350

Supplementary Table 3. Univariable and multivariable Cox regression analysis for HOMA-IR (as continuous or categorized variable) as predictor of all-cause mortality.

**Model**	***Univariable***	***Multivariable***	***Univariable***	***Multivariable***
**Predictor**	***HR (95% CI)***	***HR (95% CI)***	***HR (95% CI)***	***HR (95% CI)***
HOMA-IR (continuous)	1.04 (0.99-1.08)	1.02 (0.89-1.17)	-	-
HOMA-IR ≥ 3.91	-	-	**1.40 (1.02-1.93)**	1.46 (0.93-2.30)
Sex	1.22 (0.92-1.62)	**1.49 (1.11-2.00)**	1.22 (0.92-1.62)	**1.48 (1.10-1.98)**
Smoking	0.92 (0.61-1.37)	1.32 (0.87-1.99)	0.92 (0.62-1.37)	1.38 (0.91-2.07)
Hypertension	**1.52 (1.12-2.07)**	1.13 (0.82-1.56)	**1.52 (1.12-2.07)**	1.10 (0.80-1.52)
Age	**1.10 (1.08-1.1)**	**1.10 (1.08-1.13)**	**1.10 (1.08-1.12)**	**1.10 (1.08-1.13)**
Statin	1.19 (0.85-1.66)	1.00 (0.70-1.42)	1.19 (0.85-1.66)	0.99 (0.70-1.41)
Hb1Ac	1.09 (0.98-1.2)	1.13 (0.99-1.2)	1.09 (0.98-1.20)	1.11 (0.99-1.24)
eGFR	**0.98 (0.97-0.99)**	**0.99 (0.98-1.00)**	**0.98 (0.97-0.99)**	**0.99 (0.98-1.00**)
CHOL	1.00 (0.99-1.00)	1.00 (1.00-1.00)	1.00 (0.99-1.00)	1.00 (1.00-1.00)
hsCRP	**1.02 (1.01-1.04)**	**1.02 (1.00-1.04)**	**1.02 (1.01-1.04)**	**1.02 (1.00-1.04)**
Insulin	1.02 (0.99-1.04)	1.01 (0.94-1.08)	1.02 (0.99-1.04)	1.00 (0.97-1.03)

Univariable and multivariable (adjusted for sex, smoking, hypertension, age, BMI, Hb1Ac, eGFR, total cholesterol, and hs-CRP) hazard ratios (HR) with 95 % confidence intervals (CI) are shown. Significant predictors in the analysis are in bold.
